# Tracing Water Sources of Terrestrial Animal Populations with Stable Isotopes: Laboratory Tests with Crickets and Spiders

**DOI:** 10.1371/journal.pone.0015696

**Published:** 2010-12-31

**Authors:** Kevin E. McCluney, John L. Sabo

**Affiliations:** School of Life Sciences, Arizona State University, Tempe, Arizona, United States of America; University of Western Ontario, Canada

## Abstract

Fluxes of carbon, nitrogen, and water between ecosystem components and organisms have great impacts across levels of biological organization. Although much progress has been made in tracing carbon and nitrogen, difficulty remains in tracing water sources from the ecosystem to animals and among animals (the “*water web*”). Naturally occurring, non-radioactive isotopes of hydrogen and oxygen in water provide a potential method for tracing water sources. However, using this approach for terrestrial animals is complicated by a change in water isotopes within the body due to differences in activity of heavy and light isotopes during cuticular and transpiratory water losses. Here we present a technique to use stable water isotopes to estimate the mean mix of water sources in a population by sampling a group of sympatric animals over time. Strong correlations between H and O isotopes in the body water of animals collected over time provide linear patterns of enrichment that can be used to predict a mean mix of water sources useful in standard mixing models to determine relative source contribution. Multiple temperature and humidity treatment levels do not greatly alter these relationships, thus having little effect on our ability to estimate this population-level mix of water sources. We show evidence for the validity of using multiple samples of animal body water, collected across time, to estimate the isotopic mix of water sources in a population and more accurately trace water sources. The ability to use isotopes to document patterns of animal water use should be a great asset to biologists globally, especially those studying drylands, droughts, streamside areas, irrigated landscapes, and the effects of climate change.

## Introduction

Ecologists have long sought to trace fluxes of materials and energy within and among ecosystems [1], [2], [3], [4]. The magnitudes of these fluxes are of great importance to studies of biogeochemistry [5], physiological ecology [6], population fluctuations [7], and global change [8]. While much progress has been made in measuring fluxes of energy or nutrients to and between organisms [6], studies of water webs (as opposed to food webs) [9] are hindered by a difficulty in measuring the relative use of water sources by animal populations [10], [11]. Drinking behavior is often difficult to observe and hard to quantify. Similarly, it is difficult to determine the contribution of consumption of moist food to an animal's body water, since there is wide variation in hydration of food [12]. Cost and time-effective methods of examining water sources for a suite of animals across space or time are greatly needed.

**Figure 1:**
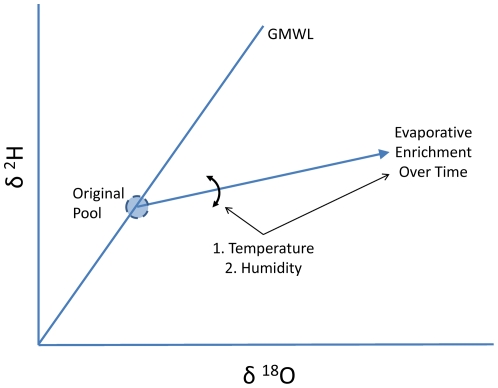
Enrichment over time from a pool of water. The original pool of water falls along the global meteoric water line (GMWL), the line representing isotope ratios of all precipitation globally. This pool then enriches in heavy isotopes over time because of higher evaporation of lighter isotopes. The rate of evaporation is influences by temperature and humidity, as is the slope of the line.

Stable water isotopes may present one possible method for tracing water sources from ecosystems to individual animals [11], [13]. Both heavy and light hydrogen (H) and oxygen (O) atoms occur naturally in all environmental water sources and vary in relative abundance in these sources due to differences in physical and chemical activity. For example, during evaporation from a pool of water, water molecules containing the lighter isotopes (^1^H and ^16^O) evaporate more readily than those containing heavy isotopes (^2^H and ^18^O), leaving a greater ratio of heavy to light isotopes in the remaining pool (Figure 1) [14], [15]. This type of change in isotope ratio is commonly known as *fractionation*.

Differences in ratios of water isotopes in various natural water pools allow for differentiation of sources used by plants. Plant physiologists and ecologists have made great use of this technique. For example, Dawson and Ehleringer [16] used natural differences in isotope ratios of groundwater and surface water, combined with measurements of xylem water, which does not fractionate, to determine that adult riparian trees used groundwater instead of surface water sources. However, all parts of a terrestrial animal are subject to differential evaporation of molecules containing H and O isotopes and thus similar methods are not possible for animals.

Sources of water used by animals often have widely separated isotope ratios. For instance, along the San Pedro River, in southeastern AZ, the trophic water available in plant leaves can be ∼90‰ δ^2^H greater than river water (δ^2^H is the ratio of ^2^H/^1^H expressed relative to an international standard, VSMOW, which is artificially referenced as 0‰ for both H and O isotopes; Text S1; Figure S1). However, methods for using water isotopes as tracers in animals are not well defined [10], [13], but this approach is promising. Wolf et al. [11] examined correlations between δ^2^H of body water and δ^13^C (^13^C/^12^C vs. a standard) of body tissues, finding that white winged doves (*Zenaida asiatica*) acquired carbon and water from cacti, but mourning doves (*Zenaida macroura*) obtained only carbon, predominantly receiving their water from some other source. This study highlighted the potential to use stable water isotopes as tracers of water consumption and demonstrated that co-occurring animal species can vary in their relative use of water sources.

In contrast to the dearth of studies examining the relative contribution of distinct water sources to the body water of animals, many studies have investigated correlations between average isotope ratios of H and O in regional precipitation and that in various tissues. For instance, Cormie et al. [17] showed that hydrogen isotope ratios of bone collagen of deer matched the average local precipitation well. Similarly, Hobson and Wassenaar [18] found correspondence between hydrogen isotope ratios of growing season precipitation and feathers of neotropical migrant songbirds. These correlations between local precipitation and body tissues have been widely used in studies of animal migration [19], [20]. However, these studies do not trace the relative contribution of distinctive sources of water to the body water of animals. Rather, these studies make use of the fact that 1) hydrogen and oxygen isotopes in water get incorporated into organic tissues of plants during photosynthesis, so these plant tissues reflect growing season precipitation [21] and 2) these organic molecules in plants get transferred up to higher trophic levels with little fractionation [22]. The body water of animals has relatively little influence on most of these organic tissues [13]. Thus, the organically bound H and O of animals reflects the organically bound H and O of plants, which in turn reflects the H and O isotopes of plant water and thus growing season precipitation. Thus, in general, organically bound H and O isotopes in animals serve as relatively good tracers of growing season precipitation, but as relatively poor tracers of distinct water sources used by animals. One notable exception is a recent model for the organically bound H and O isotope ratios of human hair [23]. However, this method only worked for the particular case of well-hydrated humans consuming a constant local water source and consistent homogenous diet across regions. Thus, the technique is likely to have relatively narrow applicability and will be unlikely to provide a method of tracing water sources in natural populations of animals with varying water sources across space or time and substantial water stress and dehydration. Methods of using body water isotopes directly to trace water sources in animals are still needed.

By examining correlations between δ^2^H and δ^13^C, Wolf et al. [11] managed to circumvent the difficult problem of fractionation of water isotopes in the body water of doves, providing some of the first evidence that water isotopes could act as tracers of water sources in animals. However, fractionation presents an obstacle to quantifying the relative use of several sources of water by animals, unless isotopic differences among sources are very large (above the natural range) [10], [11]. The most influential mechanism of animal water isotope fractionation occurs when water is evaporated from the body, typically enriching the remaining body water (i.e., increasing the δ^2^H or δ^18^O) [24], [25], however lesser changes in body water isotope ratio are also likely to come from metabolically produced water, synthesis of biochemicals, and exchange of O in water with O in CO_2_ [13], [23], [24], [25], [26]. Evaporation of water is influenced by two types of fractionation, equilibrium and kinetic, which are dependent upon environmental conditions such as temperature and the vapor pressure gradient (Figure 1). Vapor pressure, in turn, is influenced by temperature and humidity [27]. Whereas the two types of fractionation may be affected differently by these environmental factors, over time, in an open system with unsaturated air, a pool of water will enrich in heavy isotopes (Figure 1) [14].

McKechnie et al. [10] studied the potential to use ^2^H as a tracer of water sources in birds, which would require correction for ^2^H fractionation. Rock doves were equilibrated with water with a low δ^2^H and then switched to water with high δ^2^H, while varying air temperature and drinking rates via variation in the salt concentration of the water. Flow-through respirometry was used to measure the evaporative water loss of birds. Body water enriched greatly over sources in all birds (+10–50‰ δ^2^H) and the amount of enrichment was influenced by drinking rates and evaporative loss rates. The variability in enrichment caused McKechnie et al. [10] to conclude that δ^2^H alone could not be used as a general tracer for water sources in birds, without explicitly knowing information about rates of water intake and evaporative water loss for every sample taken (or unless there are very large differences between water sources: >240‰ δ^2^H). Variation in fractionation of δ^18^O, examined alone, may be even more problematic because of exchanges of O in body water with O in CO_2_ dissolved in body water. Therefore, applying isotopes as natural tracers of water sources in animals requires a different approach.

Here we describe and test a new method to estimate the mean mix of water sources in a population of animals, involving simultaneous measurement of both H and O isotopes in the body water of this population of animals. Evaporation of water from a pool leaves the remaining pool water enriched in both ^18^O and ^2^H (Figure 1). Because of relative differences in bond strength between H and O isotopes and thus differences in the activity of water molecules containing ^2^H vs ^18^O, enrichment of ^2^H occurs more quickly than ^18^O (producing a deuterium-excess) and results in characteristic lines of evaporation on a plot of δ^18^O vs. δ^2^H (Figure 1) [14]. Further, differences in humidity primarily influence the slope of the line, not the intercept of the line with the original pool of water (Figure 1) [27]. This difference in activity of the molecules is responsible for producing the global meteoric water line, a line of covariation between H and O isotope ratios that characterizes the isotope ratios of precipitation globally (Figure 1) [28]. The covariation in isotopes during evaporation from a pool also characterizes leaf water during transpiration [29], [30]. Therefore, we hypothesize that animal body water shows a similar pattern of enrichment. Isotope ratios of δ^18^O and δ^2^H within a population of animals should strongly covary and the isotopic distance between an animal's current body water and the unenriched mix of sources should be primarily determined by the relative time since source consumption (or dehydration of the individual). Humidity and temperature should only influence the slope of the line, not the intersection with the original mix of sources.

We test our hypothesis in a laboratory environment, by providing house crickets (*Acheta domesticus*) with water of a known isotope ratio, removing this water source, and collecting individuals over time. We perform these tests at three different temperatures (15°C, 25°C, and 35°C), with uncontrolled, variable, but high humidity (Table S2). At one temperature (25°C), we compare these results to results of a similar experiment in which humidity was held relatively constant and low (Table S2). We predict that lines of isotopic enrichment will be formed for each experimental run, with lines of best fit from linear regression passing through the source water. We predict that alteration of temperature and humidity will not alter the intersection of this line with the source water, although changes in slope of the line may occur (as with evaporation of a pool of water).

If validated, our technique should allow estimation of the average unenriched isotopic ratio in the average field animal (the mean isotopic mix in the population) when samples of co-occurring animals are collected across time. This estimation would allow the use of standard mixing models [6] to determine the relative contribution of multiple water sources to the body water of a group of animals. We also discuss results from a third experiment, in which field crickets (*Gryllus alogus*) and wolf spiders (*Hogna antelucana*) were switched from a depleted water source to an enriched water source under several controlled temperatures and humidities, but without controlling the mix of the two sources. Thus, we show an example of how our technique could be employed to calculate the relative contribution of these two water sources to a population of animals, but do not fully test the technique for multi-source scenarios.

## Materials and Methods

### Study Species

Single-source experiments used adult female house crickets (*Acheta domesticus*) of approximately the same age, obtained from a local pet store. The two-source experiment used lab-reared adult female damp-loving field crickets (*Gryllus alogus*), which are a southwestern US riparian specialist, and also used field-collected wolf spiders, *Hogna antelucana*, which are widely distributed in North America. *G. alogus* can be found in high density in litter dominated understory along the San Pedro River in southeastern Arizona [31]. Several studies have recently documented the ability of this species to obtain sufficient water for survival solely from greenfall (freshly fallen moist green leaves) [9], [31]. However the extent to which the species rely on other environmental sources of water, such as water from the saturated soil zone near the river, remains unknown (Text S1; Figure S1).

Large wolf spiders (*Hogna antelucana* and others) actively hunt *G. alogus* at night [9]. Recently, it has been documented that *H. antelucana* greatly alter their rates of consumption of *G. alogus* based on free water availability [9]. However, it is unknown to what extent *H. antelucana* rely on *G. alogus* for water, versus other environmental sources, such as river water from the saturated soil zone near the river (Text S1; Figure S1).

### Methods for the single-source, temperature alteration experiment

Adult female house crickets (*A. domesticus*) were separated into six cages (56×40×30 cm) with unventilated plastic lids, each containing approximately 23 crickets. Cages were housed in an environmental chamber set to 15°C, 25°C, or 35°C (depending on treatment) and a 14∶10 light∶dark cycle. These temperatures are characteristic of environmental temperatures experienced by *G. alogus* along the San Pedro river in June [31], [32]. Humidities and temperatures inside each cage were recorded at every sampling using a weather station probe (THGR122N, Oregon Scientific, ±1% RH, ±0.1°C; actual values reported in Table S2). Crickets were provided with deionized water and dry food (Teklad rodent diet 8604, Harlan, www.harlan.com) *ad libitum* as well as an egg crate for 24 hours prior to water removal. During this period, water was changed every 6 hours to reduce isotopic variation in the source water. After 24 hours, water was removed, unventilated lids were replaced with ventilated lids with mesh screen, and a glass jar of anhydrous calcium sulfate (Drierite®, W.A. Hammond Drierite Company LTD, www.drierite.com) was added to each cage to reduce humidity and speed dehydration of the crickets.

Samples of water were collected from the initial source and directly before water removal to obtain the range of possible isotopic ratios in the source water. Samples of crickets were collected directly before water removal (time 0) and 1.5, 3, 6, 12, 24, and 36 hours after water removal. One cricket was collected from each of the six cages for isotopic determination and two crickets were collected from each cage for careful gravimetric hydration measurement. All of the crickets collected for hydration were weighed before and after drying using a 5-place scale (XP205, Mettler Toledo balance with a readability of ±0.00001 g, www.mt.com). Gloves were worn throughout weighing procedures. Crickets used for hydration determination were dried at 50°C for 48 hours.

### Methods for the single-source controlled low humidity experiment

Adult female house crickets were housed for several days in an environmental chamber equipped with a space heater with a digital thermostat set to 30°C and lights set to a 14∶10 L∶D cycle. Crickets were kept in plastic containers with deionized water and food *ad libitum* as described in the single-source, temperature alteration experiment.

For this experiment, animals were then placed in individual cylindrical cages formed from chicken wire and aluminum screen, which were placed within a stainless steel chamber, all housed within a functioning environmental chamber (Text S1; Figure S2). The temperature was set to 25°C. Vapor density of influent air was maintained at approximately 3 g water/m^3^ air, by mixing variable amounts of compressed air that had been either dried or vapor-saturated. Air was dried using anhydrous calcium sulfate (Drierite®, W.A. Hammond Drierite Company LTD, www.drierite.com) or saturated by bubbling water through a heated deionized water column and mixing was accomplished using calibrated flow rotameters (Omega Model FL-3840G). The influent air flow rate was maintained at approximately 8.5 L/min. Temperature was measured by thermocouple (Type T copper and constantan wire with DiGi-Sense type T thermocouple meter, Omega Engineering, Inc., Stamford, CT) and vapor density was measured using a saturation-calibrated dewpoint hygrometer (911 Dew All™, EG&G, ±3.0°C dewpoint). Measured values were slightly different than intended settings (measured values are reported in Table S2).

Experimental runs began with 12 hours of acclimation to experimental conditions without food or water. At the end of this period, samples were collected and deionized water (the same as during rearing) was added to cages for one hour. Water was added via insertion of continuously flowing miniature water fountains through the base of the stainless steel chamber (Text S1; Figure S2). Samples of water and animals were taken before water fountains were added. After one hour of water, additional samples of animals and water were taken and water was removed. Additional samples of animals were taken 1.5, 3, 6, 12, and 24 hours post water removal. The timeline of events was similar to that of the two-source experiment, shown in Table S1.

### Methods for the two-source example experiment

This experiment was conducted in the same manner as the single-source, controlled low humidity experiment, except as noted below (Text S1; Table S1; Table S2). First, this experiment used field crickets, *G. alogus*, which were collected along the San Pedro River, reared to adulthood and bred in the lab. Moistened soil was provided for breeding. Thus, all crickets used in this experiment were reared under identical conditions from egg to adulthood. Only adult female *G. alogus*, which do not fly or stridulate (“call”), were used in experiments in order to reduce the potential influence of metabolically produced water. Spiders (*H. antelucana*) were also field captured from the same location and allowed to acclimate to the conditions of the rearing environmental chamber for at least 2 weeks. During this time they were given water *ad libitum* and fed one house cricket per week, (approximately 8–12 mm total in length). Spiders used in these experiments were maintained under these conditions for 3 weeks to 5 months.

Experimental runs were carried out on crickets at several combinations of temperature and humidity (Table 1), representative of field conditions along the San Pedro River in May and June [31], [32]. Each combination was examined in a separate experimental run with run order assigned using a modified random approach. This design was used to maximize possible interpretations in the shortest time range between runs, minimizing potential effects of changes in the source population or of population failures.

**Table 1:** Dates when each combination of temperature and humidity were tested for the two-source experiment on field crickets in 2009.

		Absolute Humidity (g/m^3^)			
		*3*	*6*	*9*	*12*
	***15***	10/23	9/4		
**Temperature (°C)**	***25***	9/24	9/14	9/18	10/8
	***35***	11/2	9/11		

Spiders were tested only at 25°C and 6 g/m^3^ on 11/18.

Each run occurred similarly to the single-source controlled low humidity experiment. As with that experiment, animals in this experiment received deionized tap water during rearing. But here, animals were switched to enriched water (by approximately 60‰ δ^2^H) for the one hour of water delivery via cricket water fountains. After this hour, cages were dried and replaced (Table S1). In this experiment, in addition to the thermocouple and dewpoint hygrometer used in the single-source controlled low humidity experiment, we also measured the temperatures and humidities of influent air using a weather station probe (THGR122N, Oregon Scientific, ±1% RH, ±0.1°C). Again, actual measurements of temperature and humidity are reported in Table S2. In all other ways, this experiment and the single-source controlled low humidity experiment were identical.

### Isotopic processing and measurement

For all experiments, samples of animals were collected in a random order, sealed in airtight vials, and frozen. Water was extracted from samples via cryogenic vacuum distillation [33], with addition of activated charcoal to remove abundant volatile organic compounds and subsequent filtering to remove particulates. Samples were then flame-sealed in glass capillary tubes until isotope analysis, which was conducted using a liquid water isotope analyzer with a PALS autosampler (DLT-100, Los Gatos Research, Inc; precision of ∼0.2‰ δ^18^O and ∼0.5‰ δ^2^H), with every two unknown samples bracketed by one of two known working standard (Los Gatos Research, Inc, standards #3 and #5) and with the first three injections discarded to reduce isotopic carryover. The validity of these methods has been verified through repeated testing (Text S1; Figure S3; Table S3). Isotope analyses of the samples from the single-source, controlled humidity experiment were conducted following similar, validated, techniques at the Colorado Plateau Stable Isotope Laboratory at Northern Arizona University.

### Statistical analysis

We employed several methods to test our hypothesis and conducted all analyses in R v2.9.0 (Text S1). Our first approach sought to examine if regression lines through isotope ratios of all collected crickets from each treatment level passed through source values. For both of the single-source experiments, we calculated simple linear regressions for each group of crickets and mean values for sources. Next we calculated the distance between the regression line and the point representing the source mean. We then broadened this approach to include 95% prediction intervals of the regression line and confidence intervals of the source, testing if there was a statistically significant difference between the cricket line and the source. Next we used ANCOVA to test for significant differences between slopes and elevations of the regression lines for each combination of temperature (15°C, 25°C, or 35°C) and humidity (variable and high vs. constant at 3 g/m^3^), followed by post-hoc comparisons between each line using Tukey's HSD, following Zar [34]. Since each sample we collected represents a separate individual, data were independent. Normality, equal variance, and linearity assumptions of ANCOVA were assessed visually using plotting tools in R v2.9.0. These assumptions were met.

For all three experiments, we examined if time since water removal had a significant effect on isotope ratios using MANOVA, with δ^2^H and δ^18^O isotopes as responses and time as the predictor, with a block representing each experimental run. To see if hydration differences might be a potential cause of any observed variation between water isotope ratios of crickets and time, we examined Spearman correlations between time and hydration of the crickets collected solely for this purpose from the single-source temperature alteration experiment. We could not directly test if hydration influenced water isotope ratios of crickets due to the losses of volatile organics during the water extraction procedure used in determining isotope ratios.

For the two-source experiment, we calculated a regression line between water isotopic measurements of crickets and another between the depleted water source available during rearing and the enriched water provided during the experiment. We then calculated the intersection of these two lines. We also calculated the intersections of the prediction intervals of the cricket line with the water source line. We used these intersection points in simple mixing models using H isotopes. The intersection of the mean regression lines represent the mean mix of sources for the entire population of crickets. The upper and lower 95% confidence levels represent the variability in the mix of sources within individual crickets, but do not indicate the error in the estimate of the population mean (Figure S4). Our current best estimate of the error in the population mean is based on the distance between the regression line and sources from the single-source experiments.

## Results

**Figure 2:**
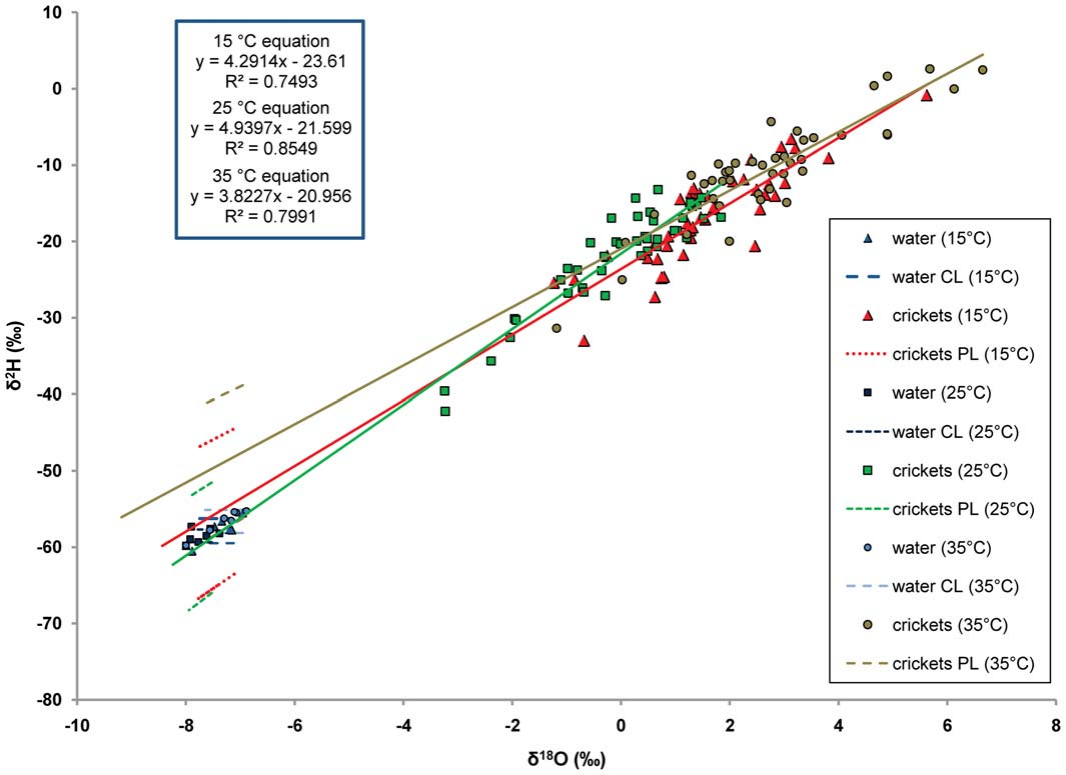
The isotope ratios of the three runs from the single-source temperature alteration experiment. All three runs experienced similar experimental conditions, with relatively high, but variable humidity (Table S2). These lines display strong and statistically significant correlations (Table 2). There are no statistical differences between the estimates of the water sources and the estimates of each run (Table 3). While there is an overall difference in slope and intercepts between all lines from the single-source experiments (Table 4), there are no statistically significant differences between any two lines (Table 5). Note: not all details are visible (e.g. the lower prediction limit for the 35°C crickets is hidden by the fitted line for the 25°C crickets).

**Figure 3:**
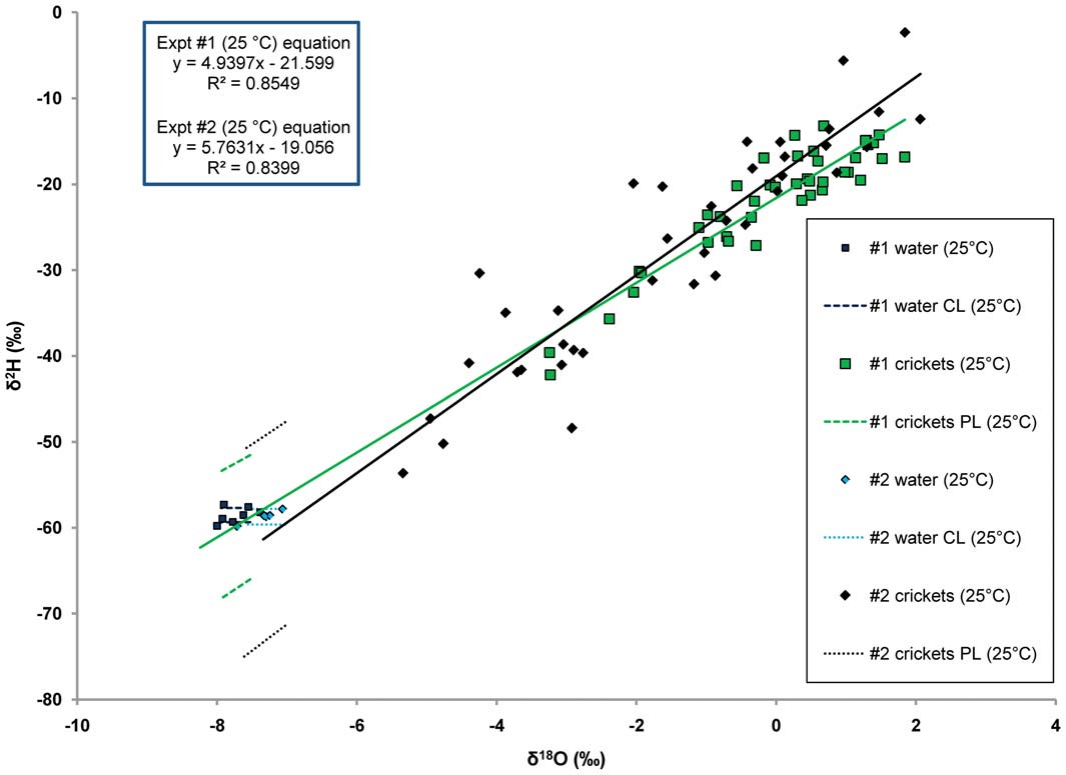
The isotope ratios of runs at 25°C from the single-source experiments. These two runs differ in humidity and experimental set-up, but not temperature. The temperature alteration experiment had relatively high, but variable humidity, whereas the controlled low humidity experiment had low and constant humidity (Table S2). These lines display strong and statistically significant correlations (Table 2). There are no statistical differences between the estimates of the water sources and the estimates of each run (Table 3). While there is an overall difference in slope and intercepts of all lines from the single-source experiments (Table 4), there are no statistically significant differences between these two lines (Table 5).

For both of the single source experiments, isotope ratios of crickets were strongly positively correlated, forming regression lines on dual isotope plots (Table 2; Figure 2; Figure 3). We found a close match between fitted cricket regression lines in both single-source experiments and the mean value of sources, with distances between these lines and sources differing by less than two parts per mil (Figure 2; Figure 3; Table 3). Additionally, there were no statistically significant differences between the estimates of the source water and the estimates of the cricket regression lines (as determined by overlapping confidence and prediction intervals; Figure 2; Figure 3; Table 3). Using ANCOVA, we found significant overall differences in the slopes of the four cricket regression lines from the single-source experiments (F_3,156_ = 6.66, p = 0.000; Table 4). However, post-hoc Tukey's comparisons did not show that any two of the slopes were significantly different from one another (Table 5).

**Table 2:** Regression statistics for each run from the single-source experiments.

*15°C, variable humidity*					
Adjusted R^2^:	0.74				
Source	Df	Sum Sq	Mean Sq	F value	p
Model	1	1241.2	1241.2	118.90	0.000
Residuals	40	417.6	10.4		
Coefficients	Estimate	Std. Error	t value	p	
(Intercept)	−23.63	0.79	−29.88	0.000	
Slope	4.27	0.39	10.90	0.000	
*25°C, variable humidity*					
Adjusted R^2^:	0.85				
Source	Df	Sum Sq	Mean Sq	F value	p
Model	1	1618.7	1618.7	235.71	0.000
Residuals	40	274.7	6.9		
Coefficients	Estimate	Std. Error	t value	p	
(Intercept)	−21.60	0.40	−53.39	0.000	
Slope	4.94	0.32	15.35	0.000	
*35°C, variable humidity*					
Adjusted R^2^:	0.79				
Source	Df	Sum Sq	Mean Sq	F value	p
Model	1	1535.2	1535.2	159.07	0.000
Residuals	40	386.1	9.7		
Coefficients	Estimate	Std. Error	t value	p	
(Intercept)	−20.96	0.95	−21.95	0.000	
Slope	3.82	0.30	12.61	0.000	
*25°C, 3g water/m^3^ air*					
Adjusted R^2^:	0.84				
Source	Df	Sum Sq	Mean Sq	F value	p
Model	1	5282.1	5282.1	188.76	0.000
Residuals	36	1007.4	28		
Coefficients	Estimate	Std. Error	t value	p	
(Intercept)	−19.05	1.05	−18.09	0.000	
Slope	5.76	0.42	13.74	0.000	

In each case, δ^2^H is the response variable and δ^18^O is the predictor.

**Table 3:** Comparison of the fitted values and upper and lower prediction levels of the δ^2^H of the cricket regression line with the values of the water source at the mean values and upper and lower confidence levels of δ^18^O of the source (CL = confidence limit, PL = prediction limit).

*15°C, variable humidity*									
		source δ^18^O	source δ^2^H			cricket δ^2^H			sig
			lower CL	mean	upper CL	lower PL	mean	upper PL	
source δ^18^O	upper CL	−7.08	−59.49	−57.87	−56.25	−63.36	−53.85	−44.34	NS
	mean	−7.43	−59.49	−57.87	−56.25	−65.09	−55.37	−45.66	NS
	lower CL	−7.79	−59.49	−57.87	−56.25	−66.82	−56.90	−46.97	NS
Distance between the mean water source and fitted values of the cricket regression line:								0.57	

*25°C, variable humidity*									
		source δ^18^O	source δ^2^H			cricket δ^2^H			sig
			lower CL	mean	upper CL	lower PL	mean	upper PL	
	upper CL	−7.53	−59.35	−58.52	−57.68	−66.03	−58.78	−51.54	NS
	mean	−7.74	−59.35	−58.52	−57.68	−67.15	−59.81	−52.48	NS
	lower CL	−7.94	−59.35	−58.52	−57.68	−68.27	−60.84	−53.41	NS
Distance between the mean water source and fitted values of the cricket regression line:								0.26	

*35°C, variable humidity*									
		source δ^18^O	source δ^2^H			cricket δ^2^H			
			lower CL	mean	upper CL	lower PL	mean	upper PL	sig
source δ^18^O	upper CL	−6.96	−58.15	−56.63	−55.11	−56.24	−47.55	−38.86	NS
	mean	−7.30	−58.15	−56.63	−55.11	−57.70	−48.86	−40.03	NS
	lower CL	−7.64	−58.15	−56.63	−55.11	−59.16	−50.18	−41.20	NS
Distance between the mean water source and fitted values of the cricket regression line:								1.97	

*25°C, 3g water/m3 air*									
		source δ^18^O	source δ^2^H			cricket δ^2^H			sig
			lower CL	mean	upper CL	lower PL	mean	upper PL	
source δ^18^O	upper CL	−7.03	−59.62	−58.71	−57.81	−71.43	−59.57	−47.71	NS
	mean	−7.32	−59.62	−58.71	−57.81	−73.23	−61.27	−49.31	NS
	lower CL	−7.62	−59.62	−58.71	−57.81	−75.05	−62.98	−50.91	NS
Distance between the mean water source and fitted values of the cricket regression line:								0.44	

Prediction intervals of the cricket line that encompass the confidence intervals of the water source indicate a lack of statistical difference between the prediction of the cricket line and the estimate of the mean of the source (NS = not significant). Also included is the distance between the mean isotopic value of the water source and the fitted cricket regression line.

All values are ‰.

**Table 4:** ANCOVA tables testing for differences in slopes and intercepts between regression lines through isotopic ratios of each run in both the single-source experiments combined.

Source	Df	Sum Sq	Mean Sq	F value	p
delta18O	1	15362.8	15362.8	1149.07	0.000
run (intercepts)	3	264.4	88.1	6.59	0.000
delta18O:run (slopes)	3	267.2	89.1	6.66	0.000
residuals	156	2085.7	13.4		

Overall, both slopes and intercepts differ between the 4 lines.

**Table 5:** Post-hoc Tukey's HSD comparisons of differences between regression lines in each run, following ANCOVA (see Table 4).

Comparison	df	q	p
25°C, variable humidity vs 25°C, 3g/m^3^	76	1.35	0.225
15°C, variable humidity vs 25°C, variable humidity	80	1.32	0.212
15°C, variable humidity vs 35°C, variable humidity	80	0.91	0.082
25°C, variable humidity vs 35°C, variable humidity	80	2.48	0.696
15°C, variable humidity vs 25°C, 3g/m^3^	76	2.35	0.650
35°C, variable humidity vs 25°C, 3g/m^3^	76	3.56	0.935

**Figure 4:**
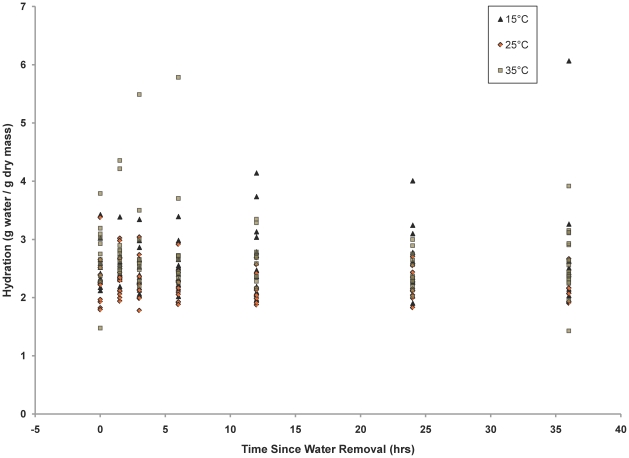
Hydration of crickets collected at each time in the single-source temperature alteration experiment. There is no significant correlation between time since water removal and hydration.

Across all experiments, we found significant variation between isotope ratios and time since water removal (MANOVA, Pillai = 0.014, F_1,317_ = 2.3, p = 0.1; Table 6). However, the single-source temperature alteration experiment revealed a lack of statistical correlation between cricket hydration and time since water removal (S = 2870355, n = 252, r = −0.08, p = 0.228). A graph shows that dehydrated crickets were present at the beginning and end of each experimental run (Figure 4).

**Table 6:** MANOVA table testing for differences in the isotope ratios of animals across collection times, including results from all 3 experiments.

Source	Df	Pillai	approx F	num Df	den Df	p
Time	1	0.018	3.284	2	358	0.039
Run	1	0.196	43.593	2	358	0.000
Residuals	359					

Significant differences were detected across collection times. However, in the single-source temperature alteration experiment, hydration was not correlated with time (S = 2870355, n = 252, r = −0.08, p = 0.228).

**Figure 5:**
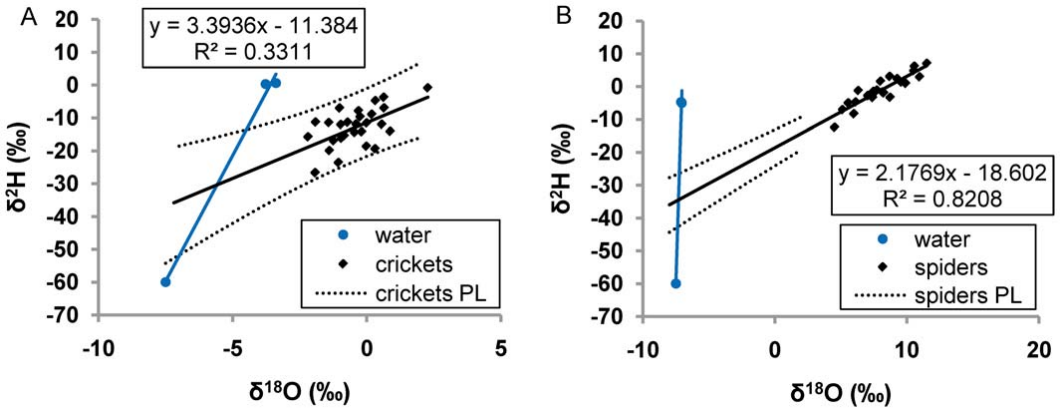
Example determinations of the mean contribution of de-ionized water to body water with two sources. Panel A shows field crickets (*G. alogus*) and panel B shows wolf spiders (*H. antelucana*), both at 25°C and 6 g/m^3^ humidity, from the two-source experiment. See Table 7 for mixing model calculations of the percentage contribution of each water source.

Following the proposed methods discussed above, in the two-source experiment we found intersection lines between the two-source water regression lines and the animal regression lines representing a mean mix of sources across all runs of 50.8% (±4.45% SE) depleted water (as opposed to enriched water; Table 7). We highlight two runs of this experiment for example purposes. First, field crickets (*G. alogus*) at 25°C and 6 g/m3 humidity were calculated to have obtained a mean of 50.9% of their body water from depleted water, with individuals varying from 21.0% to 85.4%, based on confidence intervals (Table 7; Figure 5A). Second, spiders (*H. antelucana*) at 25°C and 6 g/m^3^ humidity were calculated to have obtained a mean of 53.8% of their body water from depleted water, with individuals varying from 39.0% to 68.6%, based on confidence intervals (Table 7; Figure 5B). If we use the maximum distance between the regression line and sources from the single-source experiments of 2 parts per mil (Table 3) as an estimate of error in the mean mix of sources in the two-source experiments, it would indicate an error of approximately ±3% for the mean mix of sources in the population, in this experiment.

**Table 7:** Population mean percentage of body water obtained from the depleted water source in the two-source experiment, and lower and upper estimates of the range for individual crickets, from mixing models based on mean values of sources and intersection points between source regression lines and animal regression lines.

Type	Temperature (°C)	Humidity (g/m^3^)	Mean	Lower	Upper
Cr	15	3	61.0%	1.7%	100.0%
Cr	15	6	49.9%	12.3%	86.8%
Cr	25	3	20.5%	0.0%	50.7%
*Cr*	*25*	*6*	*50.9%*	*21.0%*	*85.4%*
Cr	25	9	62.8%	18.0%	100.0%
Cr	25	12	59.4%	11.7%	100.0%
Cr	35	3	58.7%	11.5%	100.0%
Cr	35	6	39.9%	10.2%	72.2%
*Sp*	*25*	*6*	*53.8%*	*39.0%*	*68.6%*
**mean**			**50.8%**	**13.9%**	**84.9%**
**SE**			**4.5%**	**3.9%**	**5.9%**

Lower and upper values are based on intersections of water regression line and the animal regression line prediction intervals. The italicized rows are discussed as examples in the text and graphed in Figure 5. Cr = field cricket (*G. alogus*), Sp = wolf spider (*H. antelucana*).

## Discussion

Animal ecologists have struggled to trace important fluxes of water between sources and consumers [10]. Our results suggest that water isotopes can be used as population-level tracers of animal water sources at natural abundance using the technique discussed here. Strong covariation between H and O isotope ratios of animals in the single-source experiments created regression lines that extended backwards through isotopic water sources, or very close to these sources, despite differences in temperature and humidity. Thus, if one collects body water samples from sympatric individual animals of a certain species, varying in time since consuming a similar distribution of water resources, the approximate mean mix of sources for the population should fall along a regression through the points of their δ^18^O and δ^2^H. Additionally, we speculate that the population-wide regression line could act as an integrative measure of the evaporative enrichment of the local environment and that the slope of the population-wide regression line could potentially be used to correct for the isotopic enrichment of individuals, allowing for estimation of the mix of sources for individual animals. However, this speculation requires testing.

Significant differences in slopes of regression lines in the single-source experiments provide some evidence of slight changes in fractionation associated with shifts in environmental temperature or humidity. However, we were unable to find differences between any two slopes in post-hoc comparisons. Additionally, overall differences in slope did not cause a significant difference between the predicted regression line and the values of the source (Table 3; Figure 2; Figure 3). Thus, relatively high statistical power in this experiment provided statistically significant overall differences between slopes across a shift of 20°C and considerable range in humidity, but absolute differences in slope were relatively small, having little to no effect on the validity of the technique we employed (Table 3; Figure 2; Figure 3). Even though individual animals may vary greatly in their actual enrichment, co-occurring animals of a given species are likely to experience a similar regime of factors influencing fractionation. This fractionation produces characteristic covariation between enrichment of ^2^H and ^18^O, which describes a line passing through or near the average value for the mix of sources for the population (Figure S5). Thus, such lines integrate all fractionation factors and allow for correction, without needing time series of information on temperature and humidity for each individual animal. In other words, instead of trying to determine specific values for fractionation factors, the regression techniques shown here allow for an integrated method of correcting for fractionation at the population-level that does not rely on knowing values for specific fractionation factors for individual animals. However, further investigation of the influence of temperature and humidity on the slopes and intercepts of these regression lines could be useful to verify the results presented here.

Whereas evidence for the validity of our technique appears strong, the mechanistic basis for the lines produced by animals collected in these experiments is less clear. While we found the expected significant effect of time since water removal on the isotope ratios of these crickets, we did not find a significant correlation between cricket hydration and time since water removal. There was a lack of change in the hydration of these crickets over time, with some crickets at the initial collection of the single-source temperature alteration experiment just as dehydrated as at the final collection. Additional experimentation is needed with more successful manipulation of cricket hydration. However, we note that none of the animals collected had isotope ratios very near that of the source, showing a large gap between the sources and the least enriched individual. We hypothesize that this is due to a “moving target” effect (Figure S5). This effect occurs because any individual animal is constantly enriching. When an animal consumes a new water source, it is adding a relatively small amount of the source water to a relatively large amount of enriched body water. Thus, the body water isotope ratio of each animal will move up and down near the enrichment line over time and will never approach the source values very closely. More testing of this mechanistic hypothesis is needed to achieve better confidence in the universality of the use of the described technique of correcting for enrichment.

Our analyses of two-source systems outlines the manner in which our techniques of correction for enrichment would allow determination of the relative contribution of sources to a population of animals under more realistic field conditions. Our experiment does not provide a direct test for this method in two-source systems, since the actual mix was unknown. Additionally, the estimate of the mean mix of sources for the population has no estimate of associated error. Further, repeated testing under conditions where the mean mix of sources for the population is controlled are needed in order to fully verify this technique and estimate the error in the population mean mix of sources. The confidence intervals provided by the intersection of the animal prediction lines with the water regression line estimate the variability the mix of sources within individual crickets within the population (Figure S4). We do suggest however, that until controlled two-source experiments are conducted, our single-source experiments provide some expectation of error for these methods. Since the distances between the single-source regression lines and the known sources were below 2‰ (and as low as 0.26‰), one would expect the error to be quite low in two-source scenarios. In our particular case, we calculated an error of less than ±3% in the calculation of mix of sources in the two-source experiments, based on the distances from the single source experiments. However, to verify this low error rate for multi-source scenarios, additional experiments are needed.

Our examination suggests that there are several factors that will influence precise estimates of the mix of sources: 1) the number of samples collected and 2) the perpendicularity of the water and animal regression lines. More sample collection should increase the statistical power and provide better estimates of this line. Additionally, no intersections would occur in situations where the animal regression line and the water line are parallel, invalidating the technique, so some difference in slope of these two lines is necessary. The proximity of the isotope ratio of the least enriched animals to the mix of sources and consequently, the turnover rate of body water will slightly influence the estimate of the variation in the mix of sources in individuals within the population. The closer the least-enriched animals are to the mix of sources, the narrower the prediction intervals will be at the points of intersection with the water line. Perpendicularity of the two lines may also influence estimates of this variation, since the more orthogonal the lines are, the narrower the range between the intersection of the upper and lower prediction limits of the animal regression line with the water line.

Whereas further testing is needed, our results present encouraging evidence that one can use natural abundances of stable isotopes to trace water sources in animal populations, after correcting for isotope fractionation using lines of regression on animals collected over time. This technique should be widely applicable. The spatial extent of dry lands is over ⅓ of the earth's land mass [35] and global climate change is expected to increase the variability of precipitation, with more intense storms and droughts a likely consequence [36]. Additionally, even though water limitation may not occur in all ecosystems at all times, recent evidence suggests that water may be limiting in all ecosystems, even rainforests, at some time, in some years [37] and that this variability in water availability may influence physiology, behavior, species interactions, and diversity [9], [31], [38], [39], [40]. Thus, it is exceedingly important that ecologists, resource managers, and the public understand the potential impacts of changing water availability and declining surface waters. The methods developed here should provide tools to examine patterns of water use in an ecological community and help to predict the potential impacts of changes in water resources.

## Supporting Information

Text S1 **Additional methodological details and supporting isotope values from field collections**.
(DOC)

Figure S1 **Results from field collections of sources, crickets, and spiders.** Proximal samples are those collected within 10 m of the flowing river and distal samples were collected greater than 50 m from the flowing river.
(JPG)

Figure S2 **Experimental apparatus design for the single‐source controlled low humidity experiment and the two‐source experiment**. Panel A shows a top view, panel B shows a side view, and panel C shows a close‐up of a miniature water fountain.
(TIF)

Figure S3 **Effects of different amounts of activated charcoal addition on the isotope ratio of extracted crickets.** Even a small amount of charcoal reduces the influence of volatile organic compounds on isotope ratios.
(TIF)

Figure S4 **Comparison of two hypothetical animal populations in a two‐source system. **In both cases, the mean mix for the population is 50% of each source. However, the left panel shows a population experiencing lower inter‐individual variability in source use and the right panel shows high.
(TIF)

Figure S5 **Hypothetical graph of the factors influencing the isotope ratio of individual animals, displayed as vectors**. Black arrows indicate the relative magnitude of influence of each driver of the isotope ratio of body water of individuals.
(TIF)

Table S1 **Timeline of activities for each run of the two‐source experiment**.
(DOC)

Table S2 **Planned vs. actual temperature and humidity for each run of each experiment**.
(DOC)

Table S3 **Results of tests of extraction and processing of water samples for isotope analysis. Raw data are reported for each category of sampling**.
(DOC)
