# Supplementary material for: Tracing Water Sources of Terrestrial Animal Populations with Stable Isotopes: Laboratory Tests with Crickets and Spiders
Source: PLoS One. 2010 Dec 31;5(12):e15696. doi: 10.1371/journal.pone.0015696 (PMC3013119; doi:10.1371/journal.pone.0015696)
Supplement: Text S1 — Additional methodological details and supporting isotope values from field collections. (DOC) [file pone.0015696.s001.doc]

### Text S1

#### Preliminary Field Collections of Isotopes

In the dry season of 2004, we collected samples of possible water sources and arthropods proximal (< 10 m) to a flowing reach of the San Pedro River and distal (>50 m) to the flowing river. Each sample was a combination of multiple individuals, thus we do not have individual estimates of isotope ratios. All collections were made under cottonwood-willow gallery forest. Samples were collected by hand and added to airtight vials with polyseal caps and frozen until analysis. Analysis took place at the Stable Isotope Ratio Facility for Environmental Research (SIRFER) at the University of Utah.

Three results from this work are relevant here: 1) Use of river water is apparent in riparian consumers, 2) some consumers seem to more heavily rely on river water, and 3) consumer body water is enriched compared to sources, suggesting that fractionation may confound estimation of relative use of sources. More specifically, an affect of surface water on crickets is apparent in δ^2^H, with data showing significantly decreased values when crickets are near river water (t = 2.369, df = 13.3, p = 0.034). However, this difference does appear small (Figure S1, proximal vs distal crickets). Differences between spiders near and far from the river appear larger (Figure S1, proximal vs distal spiders), though the differences are not significant, perhaps because of small sample sizes (n = 3). Moreover, comparison of δ^2^H and δ^18^O values for an herbivore (grasshopper) with sources (vegetation) along a dry reach, suggests enrichment (data not shown), where the grasshopper has a heavier isotope ratio than all sources.

#### Additional procedural details of single-source constant low humidity experiment and two-source experiment

We allowed the chamber (Figure S2) to acclimate to new temperature settings for at least 24 hours prior to any experimental run. Humidity settings on the chamber were altered at least 1 hour before animals were added. For each run, we performed a series of activities shown in Table S1 for the two source experiment. The single-source low humidity experiment occurred similarly. Due to the time required for performing activities at 10 am, actual experimental initiation would often occur 10-25 minutes later (between 10:10 and 10:25 am). All subsequent times would be adjusted to preserve consistency among subsequent activities. Additionally, activities at 11 am often resulted in the chamber not being dry and sealed until approximately 11:40 am; however, we did not alter collection times. Thus all experimental runs have less well controlled moisture conditions between time 0 and time 1.5 collections. Temperatures and humidities were slightly different than planned in all three experiments. We report actual temperatures and humidities in Table S2.

#### Testing of Extraction and Processing Techniques

Extraction of water from crickets, spiders, and other arthropods via common procedures of cryogenic vacuum distillation also collects volatile organic compounds in the final sample, which are visible and have a distinctive odor. To reduce the effects of these compounds on isotope analysis, we added activated charcoal to the sample collection test tube and used a heat gun to dry out the charcoal prior to beginning extraction. At the end of extraction, we allowed the extracted sample to defrost and provided at least 10 mins for the activated charcoal to reduce these compounds. We then filtered the sample using Spin-X centrifuge tube filters and flame sealed the resulting sample in a 100 μL capillary tube until analysis.

To test if addition of activated charcoal would reduce the effect of volatile organics on the measured isotope ratio, we examined the effects of varying amounts of charcoal addition on the isotope ratios of extracted crickets which were raised under consistent conditions in the lab. In this test we were using a Thermo-Finnigan MAT 253 mass spectrometer coupled to a TC/EA and an autosampler to determine isotope ratios of water samples. We found that adding even small amounts of activated charcoal substantially reduces the effects of these volatile organic compounds (Figure S3). To test for errors in water extraction and processing we examined differences between de-ionized tap water and de-ionized tap water that was run through the entire extraction process, including charcoal addition, filtration, and flame sealing. This extraction process produced results that differed from the de-ionized by amounts within the range of measurement error (Table S3).

R code for statistical analyses

setwd("") ## set to a local directory

library(stats)

library(stats4)

##sink(file = "Routput.doc")

##Loading data

idata = read.csv("IDataCombined.csv", header=T, sep=",")

##subsets

water <- subset(idata, type == "water")

animals <- subset(idata, type != "water")

hcrickets <- subset(idata, type == "hcricket")

##EXPERIMENTS #1 AND #2 ONLY (SINGLE-SOURCE)

##create storage data frame

match1n2 <- data.frame(run=character(0), wDmean=numeric(0), wDlowerci=numeric(0), wDupperci=numeric(0), w18Omean=numeric(0), w18Olowerci=numeric(0), w18Oupperci=numeric(0), cmeanint=numeric(0), cmeanslope=numeric(0), fitmean=numeric(0), lwrmean=numeric(0), uprmean=numeric(0), fitlwr=numeric(0), lwrlwr=numeric(0), uprlwr=numeric(0), fitupr=numeric(0), lwrupr=numeric(0), uprupr=numeric(0))

##MEAN SOURCE VALUES AND CI

Wr1 <- subset(water, run. == "1")

lmwr1D <- lm(deltaD ~1, Wr1)

summary(lmwr1D)

wr1Dci <- confint(lmwr1D)

lmwr118O <- lm(delta18O ~1, Wr1)

summary(lmwr118O)

wr118Oci <- confint(lmwr118O)

Wr11 <- subset(water, run. == "11")

lmwr11D <- lm(deltaD ~1, Wr11)

summary(lmwr11D)

wr11Dci <- confint(lmwr11D)

lmwr1118O <- lm(delta18O ~1, Wr11)

summary(lmwr1118O)

wr1118Oci <- confint(lmwr1118O)

Wr12 <- subset(water, run. == "12")

lmwr12D <- lm(deltaD ~1, Wr12)

summary(lmwr12D)

wr12Dci <- confint(lmwr12D)

lmwr1218O <- lm(delta18O ~1, Wr12)

summary(lmwr1218O)

wr1218Oci <- confint(lmwr1218O)

Wr13 <- subset(water, run. == "13")

lmwr13D <- lm(deltaD ~1, Wr13)

summary(lmwr13D)

wr13Dci <- confint(lmwr13D)

lmwr1318O <- lm(delta18O ~1, Wr13)

summary(lmwr1318O)

wr1318Oci <- confint(lmwr1318O)

##CRICKET REGRESSIONS AND PI

Cr1 <- subset(hcrickets, run. == "1")

lmcr1 <- lm(deltaD ~ delta18O, Cr1)

anova(lmcr1)

summary(lmcr1)

capture.output(anova(lmcr1), file = "lmcr1anova.txt")

capture.output(summary(lmcr1), file = "lmcr1sum.txt")

new <- data.frame(delta18O = c(as.numeric(lmwr118O$coefficients), wr118Oci[1,1], wr118Oci[1,2]))

Cr1PI <- data.frame(predict(lmcr1, newdata = new, interval = "prediction"))

##new <- data.frame(delta18O = seq(-9, 2, 0.5))

##cr1pred <- data.frame(cbind(new, predict(lm125C, newdata = new, interval = "prediction")))

##cr1predlwrlm <- lm(lwr ~ delta18O, cr1pred)

##cr1preduprlm <- lm(upr ~ delta18O, cr1pred)

##combine various data into a row of data

datatemp <- cbind("1", as.numeric(lmwr1D$coefficients), wr1Dci[1,1], wr1Dci[1,2], as.numeric(lmwr118O$coefficients), wr118Oci[1,1], wr118Oci[1,2], as.numeric(lmcr1$coefficients[1]), as.numeric(lmcr1$coefficients[2]), Cr1PI$fit[1], Cr1PI$lwr[1], Cr1PI$upr[1], Cr1PI$fit[2], Cr1PI$lwr[2], Cr1PI$upr[2], Cr1PI$fit[3], Cr1PI$lwr[3], Cr1PI$upr[3])

##fill in data in a new row

match1n2 <- rbind(match1n2, datatemp)

Cr11 <- subset(hcrickets, run. == "11")

lmcr11 <- lm(deltaD ~ delta18O, Cr11)

anova(lmcr11)

summary(lmcr11)

capture.output(anova(lmcr11), file = "lmcr11anova.txt")

capture.output(summary(lmcr11), file = "lmcr11sum.txt")

new <- data.frame(delta18O = c(as.numeric(lmwr1118O$coefficients), wr1118Oci[1,1], wr1118Oci[1,2]))

Cr11PI <- data.frame(predict(lmcr11, newdata = new, interval = "prediction"))

##combine various data into a row of data

datatemp <- cbind("11", as.numeric(lmwr11D$coefficients), wr11Dci[1,1], wr11Dci[1,2], as.numeric(lmwr1118O$coefficients), wr1118Oci[1,1], wr1118Oci[1,2], as.numeric(lmcr11$coefficients[1]), as.numeric(lmcr11$coefficients[2]), Cr11PI$fit[1], Cr11PI$lwr[1], Cr11PI$upr[1], Cr11PI$fit[2], Cr11PI$lwr[2], Cr11PI$upr[2], Cr11PI$fit[3], Cr11PI$lwr[3], Cr11PI$upr[3])

##fill in data in a new row

match1n2 <- rbind(match1n2, datatemp)

Cr12 <- subset(hcrickets, run. == "12")

lmcr12 <- lm(deltaD ~ delta18O, Cr12)

anova(lmcr12)

summary(lmcr12)

capture.output(anova(lmcr12), file = "lmcr12anova.txt")

capture.output(summary(lmcr12), file = "lmcr12sum.txt")

new <- data.frame(delta18O = c(as.numeric(lmwr1218O$coefficients), wr1218Oci[1,1], wr1218Oci[1,2]))

Cr12PI <- data.frame(predict(lmcr12, newdata = new, interval = "prediction"))

##combine various data into a row of data

datatemp <- cbind("12", as.numeric(lmwr12D$coefficients), wr12Dci[1,1], wr12Dci[1,2], as.numeric(lmwr1218O$coefficients), wr1218Oci[1,1], wr1218Oci[1,2], as.numeric(lmcr12$coefficients[1]), as.numeric(lmcr12$coefficients[2]), Cr12PI$fit[1], Cr12PI$lwr[1], Cr12PI$upr[1], Cr12PI$fit[2], Cr12PI$lwr[2], Cr12PI$upr[2], Cr12PI$fit[3], Cr12PI$lwr[3], Cr12PI$upr[3])

##fill in data in a new row

match1n2 <- rbind(match1n2, datatemp)

Cr13 <- subset(hcrickets, run. == "13")

lmcr13 <- lm(deltaD ~ delta18O, Cr13)

anova(lmcr13)

summary(lmcr13)

capture.output(anova(lmcr13), file = "lmcr13anova.txt")

capture.output(summary(lmcr13), file = "lmcr13sum.txt")

new <- data.frame(delta18O = c(as.numeric(lmwr1318O$coefficients), wr1318Oci[1,1], wr1318Oci[1,2]))

Cr13PI <- data.frame(predict(lmcr13, newdata = new, interval = "prediction"))

##combine various data into a row of data

datatemp <- cbind("13", as.numeric(lmwr13D$coefficients), wr13Dci[1,1], wr13Dci[1,2], as.numeric(lmwr1318O$coefficients), wr1318Oci[1,1], wr1318Oci[1,2], as.numeric(lmcr13$coefficients[1]), as.numeric(lmcr13$coefficients[2]), Cr13PI$fit[1], Cr13PI$lwr[1], Cr13PI$upr[1], Cr13PI$fit[2], Cr13PI$lwr[2], Cr13PI$upr[2], Cr13PI$fit[3], Cr13PI$lwr[3], Cr13PI$upr[3])

##fill in data in a new row

match1n2 <- rbind(match1n2, datatemp)

## on the prediction intervals, the first part of the name refers to the delta D of the crickets and the second part refers to the 18O of the source

names(match1n2)[1:18] <- c('run', 'wDmean', 'wDlowerci', 'wDupperci', 'w18Omean', 'w18Olowerci', 'w18Oupperci', 'cmeanint', 'cmeanslope', 'fitmean', 'lwrmean', 'uprmean', 'fitlwr', 'lwrlwr', 'uprlwr', 'fitupr', 'lwrupr', 'uprupr')

write.csv(match1n2, file = "1n2Match.csv")

##calculate differences between source point and cricket line in excel

##ANCOVA (differences between 4 runs)

hcrickets$run. <- as.factor(hcrickets$run.)

ancova1n2 <- lm(deltaD ~ delta18O + run. + delta18O:run., hcrickets)

capture.output(anova(ancova1n2), file = "ancova1n2.txt")

plot(ancova1n2) ##graphically assumptions roughly matched 3/16/10

## POST-HOC TESTS OF DIFFERENCE IN SLOPES BASED ON ZAR 1999

#each group data

hcrickets1 <- subset(hcrickets, run. == "1")

hcrickets11 <- subset(hcrickets, run. == "11")

hcrickets12 <- subset(hcrickets, run. == "12")

hcrickets13 <- subset(hcrickets, run. == "13")

#fit a lm to each group separately

fit1 <- lm(hcrickets1$deltaD ~ hcrickets1$delta18O)

fit11 <- lm(hcrickets11$deltaD ~ hcrickets11$delta18O)

fit12 <- lm(hcrickets12$deltaD ~ hcrickets12$delta18O)

fit13 <- lm(hcrickets13$deltaD ~ hcrickets13$delta18O)

#extract estimates of coefficients

s1 <- summary(fit1)$coefficients

s11 <- summary(fit11)$coefficients

s12 <- summary(fit12)$coefficients

s13 <- summary(fit13)$coefficients

#create anova tables

a1 <- anova(fit1)

a11 <- anova(fit11)

a12 <- anova(fit12)

a13 <- anova(fit13)

##slopes

s1[2,1]

s11[2,1]

s12[2,1]

s13[2,1]

## Ordered magnitudes of slopes 12<13<11<1

## difference between slopes

db1.12 <- (s1[2,1]-s12[2,1])

db1.13 <- (s1[2,1]-s13[2,1])

db1.11 <- (s1[2,1]-s11[2,1])

db11.13 <- (s11[2,1]-s13[2,1])

db11.12 <- (s11[2,1]-s12[2,1])

db13.12 <- (s13[2,1]-s12[2,1])

## various equations relevant to calculations

## total SS = resid SS + regress SS

## regress SS = b * sum xy

## sum xy = regress SS / b

## b = sum xy/sum x2

## sum x2 = sum xy/b

## sum y2 = total SS

## regression SS = (sum xy)2 / sum x2

## MSE = s2y.x

## residual standard error = standard error of regression = standard error of estimate = sqrt(MSE) = sy.x

## pooled s2yx = pooled MSE = sum of MSE

s2yx1.12 <- (a1[2,3] + a12[2,3])

s2yx1.13 <- (a1[2,3] + a13[2,3])

s2yx1.11 <- (a1[2,3] + a11[2,3])

s2yx11.13 <- (a11[2,3] + a13[2,3])

s2yx11.12 <- (a11[2,3] + a12[2,3])

s2yx13.12 <- (a13[2,3] + a12[2,3])

## sum xy

sxy1 <- (a1[1,2] / s1[2,1])

sxy11 <- (a11[1,2] / s11[2,1])

sxy12 <- (a12[1,2] / s12[2,1])

sxy13 <- (a13[1,2] / s13[2,1])

## sum x2

sx21 <- (sxy1 / s1[2,1])

sx211 <- (sxy11 / s11[2,1])

sx212 <- (sxy12 / s12[2,1])

sx213 <- (sxy13 / s13[2,1])

## regression SS – just a double check

Regtest1 <- (((sxy1)*(sxy1)) / sx21)

## SE (for this test)

SE1.12 <- (sqrt((s2yx1.12/2)*((1/sx21)+(1/sx212))))

SE1.13 <- (sqrt((s2yx1.13/2)*((1/sx21)+(1/sx213))))

SE1.11 <- (sqrt((s2yx1.11/2)*((1/sx21)+(1/sx211))))

SE11.13 <- (sqrt((s2yx11.13/2)*((1/sx211)+(1/sx213))))

SE11.12 <- (sqrt((s2yx11.12/2)*((1/sx211)+(1/sx212))))

SE13.12 <- (sqrt((s2yx13.12/2)*((1/sx213)+(1/sx212))))

##pooled df

dfp1.12 <- fit1$df.residual+ fit12$df.residual

dfp1.13 <- fit1$df.residual+ fit13$df.residual

dfp1.11 <- fit1$df.residual+ fit11$df.residual

dfp11.13 <- fit11$df.residual+ fit13$df.residual

dfp11.12 <- fit11$df.residual+ fit12$df.residual

dfp13.12 <- fit13$df.residual+ fit12$df.residual

##test statistic = q

q1.12 <- (db1.12/SE1.12)

q1.13 <- (db1.13/SE1.13)

q1.11 <- (db1.11/SE1.11)

q11.13 <- (db11.13/SE11.13)

q11.12 <- (db11.12/SE11.12)

q13.12 <- (db13.12/SE13.12)

##p-values

p1.12 <- ptukey(q1.12, 4, dfp1.12)

p1.13 <- ptukey(q1.13, 4, dfp1.13)

p1.11 <- ptukey(q1.11, 4, dfp1.11)

p11.13 <- ptukey(q11.13, 4, dfp11.13)

p11.12 <- ptukey(q11.12, 4, dfp11.12)

p13.12 <- ptukey(q13.12, 4, dfp13.12)

dfp1.12

dfp1.13

dfp1.11

dfp11.13

dfp11.12

dfp13.12

q1.12

q1.13

q1.11

q11.13

q11.12

q13.12

p1.12

p1.13

p1.11

p11.13

p11.12

p13.12

##ALL THREE EXPERIMENTS

##MANOVA

Y <- cbind(animals$deltaD, animals$delta18O)

ManovaTime1n2n3 <- manova(Y ~ animals$time + animals$run.)

summary(ManovaTime1n2n3)

capture.output(summary(ManovaTime1n2n3), file = "ManovaTime1n2n3.txt")

##import text files into excel, placing appropriate dividers

##EXPERIMENT #3 (TWO-SOURCES)

##Storage data frame

calcs3 <- data.frame(run=character(0), wintercept=numeric(0), wslope=numeric(0), aintercept=numeric(0), aslope=numeric(0), delta18Omeanintersect=numeric(0), deltaDmeanintersect=numeric(0), mixmean=numeric(0), delta18Omeanlowerintersect=numeric(0), deltaDmeanlowerintersect=numeric(0), mixmeanlower=numeric(0), delta18Omeanupperintersect=numeric(0), deltaDmeanupperintersect=numeric(0), mixmeanupper=numeric(0))

##WATER REGRESSIONS AND CI

i <- 2

while (i < 11) {

print(' ', quote = F)

label <- paste('Water Run # ', i)

print(label)

W <- subset(water, run. == i)

lm3w <- lm(deltaD ~ delta18O, W)

print(anova(lm3w))

print(summary(lm3w))

new <- data.frame(delta18O = seq(-9, 2, 0.5))

WCI <- data.frame(cbind(new, predict(lm3w, newdata = new, interval = "confidence")))

WCIlwrlm <- lm(lwr ~ delta18O, WCI)

WCIuprlm <- lm(upr ~ delta18O, WCI)

##ANIMAL REGRESSIONS AND PI

print(' ', quote = F)

label <- paste('Animal Run # ', i)

print(label)

label <- paste(label, '.txt')

A <- subset(animals, run. == i)

lm3a <- lm(deltaD ~ delta18O, A)

print(anova(lm3a))

print(summary(lm3a))

API <- data.frame(cbind(new, predict(lm3a, newdata = new, interval = "prediction")))

capture.output(API, file = label)

APIlwrlm <- lm(lwr ~ delta18O, API)

APIuprlm <- lm(upr ~ delta18O, API)

##INTERSECTION BETWEEN TWO LINES

##water mean-animal mean

intersectxmeanmean <- ((as.numeric(lm3a$coefficients[1])- as.numeric(lm3w$coefficients[1])) / (as.numeric(lm3w$coefficients[2])- as.numeric(lm3a$coefficients[2])))

intersectymeanmean <- (as.numeric(lm3w$coefficients[2])*intersectxmeanmean + as.numeric(lm3w$coefficients[1]))

##water mean-animal lower

intersectxmeanlower <- ((as.numeric(APIlwrlm$coefficients[1]) - as.numeric(lm3w$coefficients[1])) / (as.numeric(lm3w$coefficients[2])- as.numeric(APIlwrlm$coefficients[2])))

intersectymeanlower <- (as.numeric(lm3w$coefficients[2])*intersectxmeanlower + as.numeric(lm3w$coefficients[1]))

##water mean-animal upper

intersectxmeanupper <- ((as.numeric(APIuprlm$coefficients[1]) - as.numeric(lm3w$coefficients[1])) / (as.numeric(lm3w$coefficients[2])- as.numeric(APIuprlm$coefficients[2])))

intersectymeanupper <- (as.numeric(lm3w$coefficients[2])*intersectxmeanupper + as.numeric(lm3w$coefficients[1]))

##MIXING MODEL CALCULATIONS

E <- subset(W, time == "-1" | time == "0")

##water mean-animal mean

proporDImeanmean <- (intersectymeanmean - mean(E$deltaD)) / (-60 - mean(E$deltaD))

##water mean-animal lower

proporDImeanlower <- (intersectymeanlower - mean(E$deltaD)) / (-60 - mean(E$deltaD))

##water mean-animal upper

proporDImeanupper <- (intersectymeanupper - mean(E$deltaD)) / (-60 - mean(E$deltaD))

wintercept <- as.numeric(lm3w$coefficients[1])

wslope <- as.numeric(lm3w$coefficients[2])

aintercept <- as.numeric(lm3a$coefficients[1])

aslope <- as.numeric(lm3a$coefficients[2])

datatemp <- cbind(i, wintercept, wslope, aintercept, aslope, intersectxmeanmean, intersectymeanmean, proporDImeanmean, intersectxmeanlower, intersectymeanlower, proporDImeanlower, intersectxmeanupper, intersectymeanupper, proporDImeanupper)

calcs3 <- rbind(calcs3, datatemp)

i <- i+1

} ## end of while loop

write.csv(calcs3, file = "calc3.csv")

##EXPERIMENT #1

##HYDRATION VS TIME CORRELATIONS (spearman)

##Loading data

hdata = read.csv("AllHydrationData.csv", header=T, sep=",")

names(hdata)

cor.test(hdata$time, hdata$Hydration, method = "spearm")
