# Supplementary figures and images for: Tracing Water Sources of Terrestrial Animal Populations with Stable Isotopes: Laboratory Tests with Crickets and Spiders
Source: PLoS One. 2010 Dec 31;5(12):e15696. doi: 10.1371/journal.pone.0015696 (PMC3013119; doi:10.1371/journal.pone.0015696)

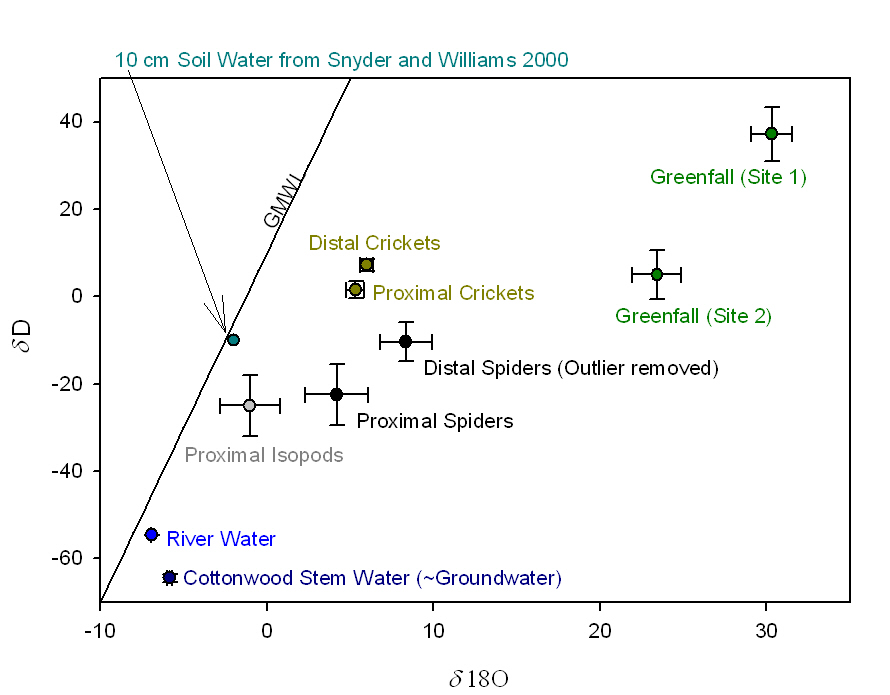

Supplement: Figure S1 — Results from field collections of sources, crickets, and spiders. Proximal samples are those collected within 10 m of the flowing river and distal samples were collected greater than 50 m from the flowing river. (JPG) [file pone.0015696.s002.jpg]

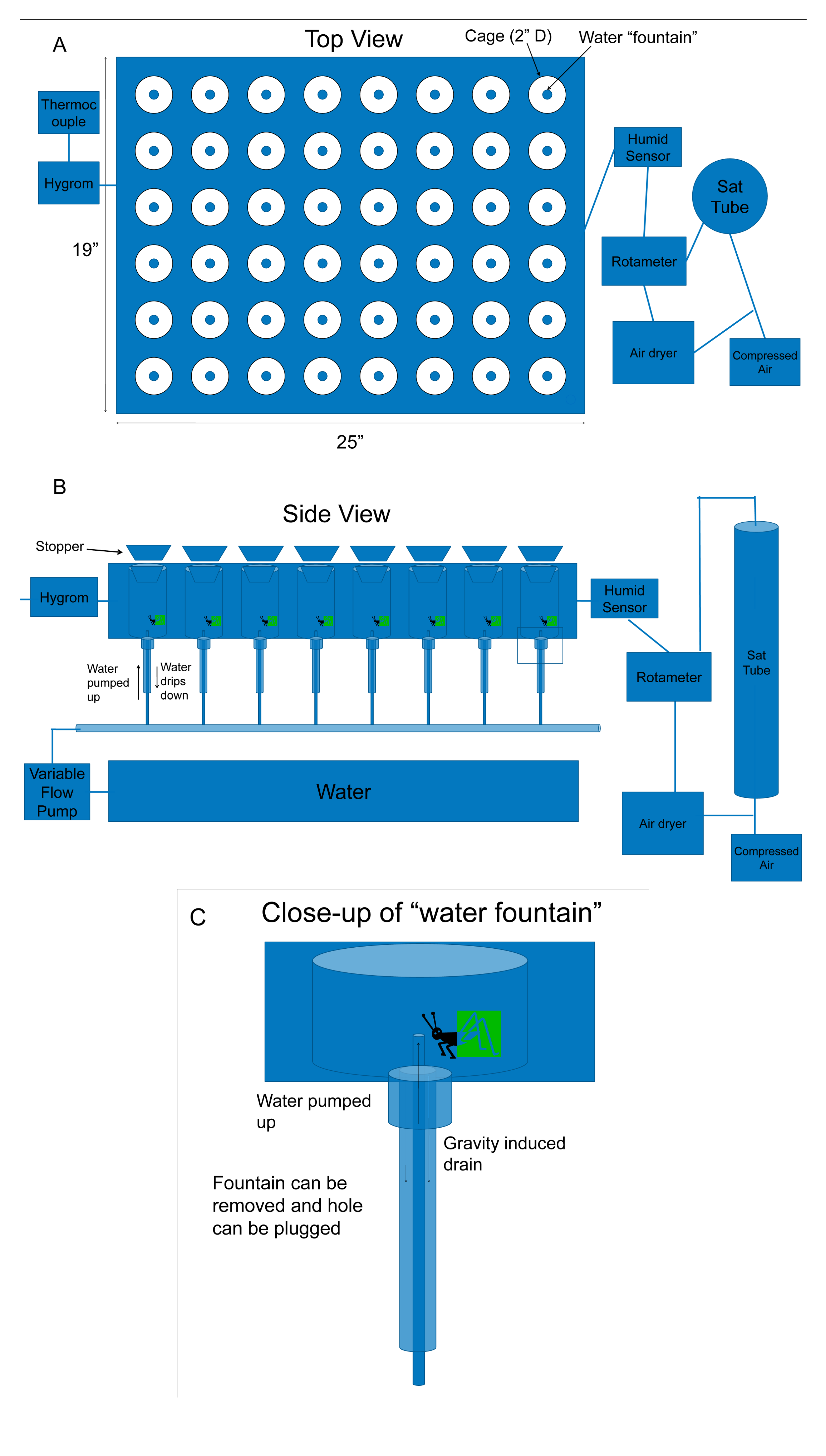

Supplement: Figure S2 — Experimental apparatus design for the single‐source controlled low humidity experiment and the two‐source experiment. Panel A shows a top view, panel B shows a side view, and panel C shows a close‐up of a miniature water fountain. (TIF) [file pone.0015696.s003.tif]

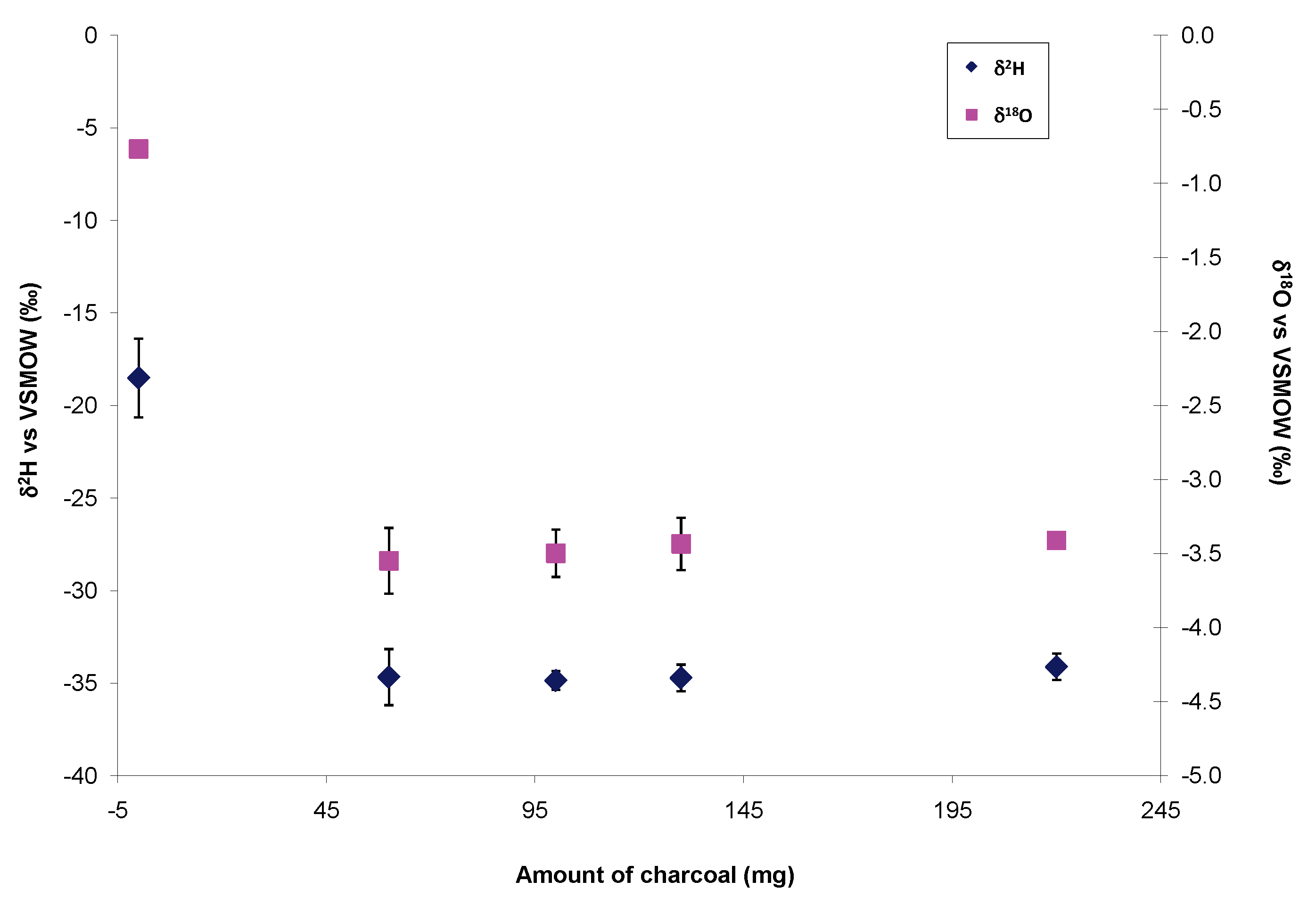

Supplement: Figure S3 — Effects of different amounts of activated charcoal addition on the isotope ratio of extracted crickets. Even a small amount of charcoal reduces the influence of volatile organic compounds on isotope ratios. (TIF) [file pone.0015696.s004.tif]

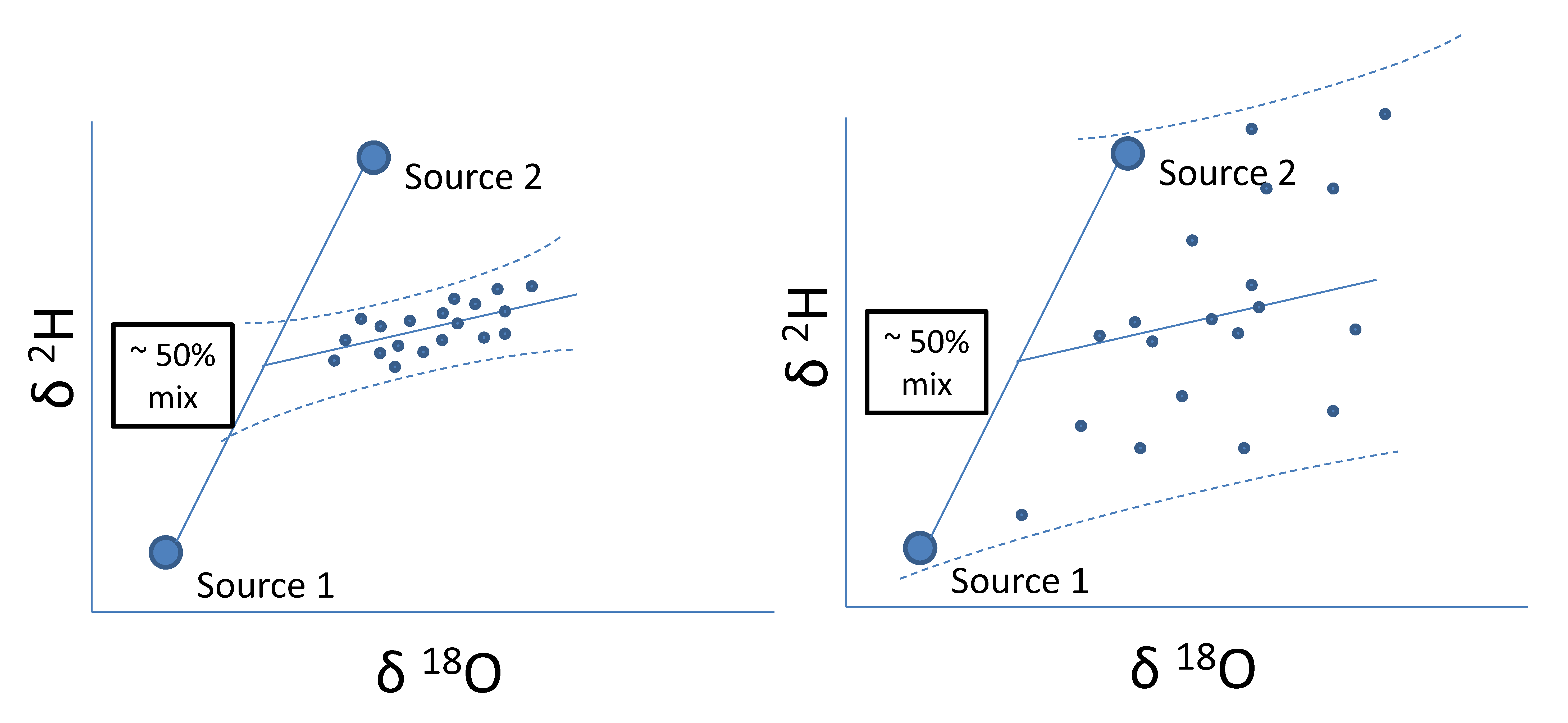

Supplement: Figure S4 — Comparison of two hypothetical animal populations in a two‐source system. In both cases, the mean mix for the population is 50% of each source. However, the left panel shows a population experiencing lower inter‐individual variability in source use and the right panel shows high. (TIF) [file pone.0015696.s005.tif]

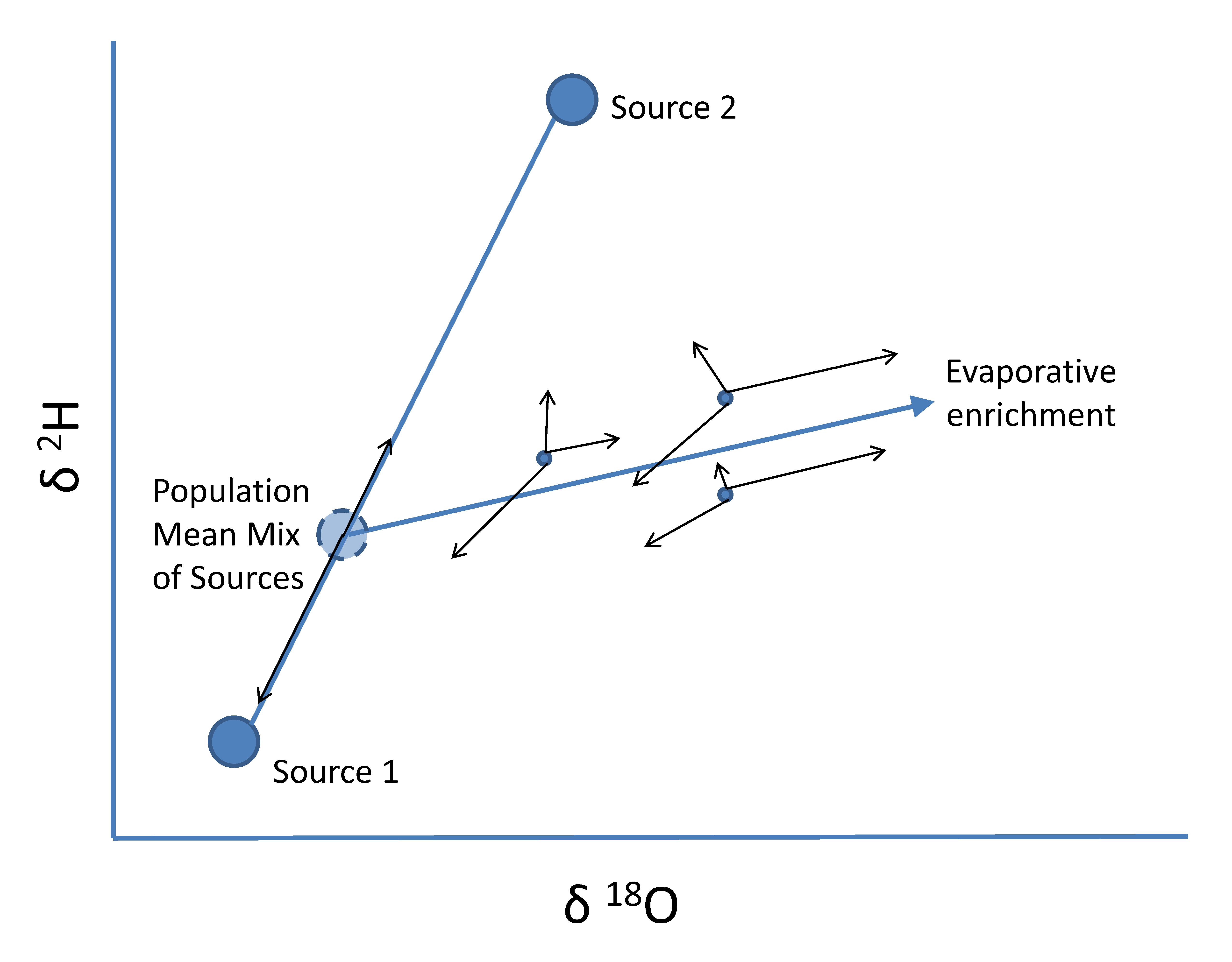

Supplement: Figure S5 — Hypothetical graph of the factors influencing the isotope ratio of individual animals, displayed as vectors. Black arrows indicate the relative magnitude of influence of each driver of the isotope ratio of body water of individuals. (TIF) [file pone.0015696.s006.tif]
