# Supplementary material for: Tracing Water Sources of Terrestrial Animal Populations with Stable Isotopes: Laboratory Tests with Crickets and Spiders
Source: PLoS One. 2010 Dec 31;5(12):e15696. doi: 10.1371/journal.pone.0015696 (PMC3013119; doi:10.1371/journal.pone.0015696)
Supplement: Table S1 — Timeline of activities for each run of the two‐source experiment. (DOC) [file pone.0015696.s007.doc]

| Table S1. Timeline of activities for each run of the two-source experiment. | |
| --- | --- |
| Time | Activity |
| 2:30 PM | Take water away from animals in preparation room |
| 9:00 PM | Add de-ionized tap water to animals in preparation room |
| 10:00 PM | Remove water from animals in preparation room and move them to the experimental chamber |
| 10:00 AM | Collect t-1 samples of animals |
| 10:00 AM | Add ^2^H enriched de-ionized tap water to animals in experimental chamber via cricket water fountains and collect samples of water |
| 11:00 AM | Collect t0 samples of water, remove water, collect t0 samples of animals. Remove cages, dry with towel, and replace cages. Seal chamber. |
| 12:30 PM | collect t1.5 samples of animals |
| 2:00 PM | collect t3 samples of animals |
| 5:00 PM | collect t6 samples of animals |
| 11:00 PM | collect t12 samples of animals |
| 11:00 AM | collect t24 samples of animals |
