# Supplementary material for: Tracing Water Sources of Terrestrial Animal Populations with Stable Isotopes: Laboratory Tests with Crickets and Spiders
Source: PLoS One. 2010 Dec 31;5(12):e15696. doi: 10.1371/journal.pone.0015696 (PMC3013119; doi:10.1371/journal.pone.0015696)
Supplement: Table S2 — Planned vs. actual temperature and humidity for each run of each experiment. (DOC) [file pone.0015696.s008.doc]

| Table S2. Planned vs. actual temperature and humidity for each run of each experiment. | | | | |
| --- | --- | --- | --- | --- |
|  | *Planned* | | *Actual* | |
| Species | temperature (°C) | abs humidity (g/m^3^) | temperature (°C) | abs humidity (g/m^3^) |
| Single-source, temperature alteration experiment | | | | |
| *A. domesticus* | 15 | Variable | 15.5 | Started at 7.29 and declined to 6.90 |
| *A. domesticus* | 25 | Variable | 23 | Started at 12.38 and declined to 7.31 |
| *A. domesticus* | 35 | Variable | 32 | Started at 12.20 and declined to 10.15 |
| Single-source, controlled low humidity experiment | | | | |
| *A. domesticus* | 25 | 3 | 23 | ~ 3 |
| Two-source experiment | | | | |
| *G. alogus* | 15 | 3 | 16.5 | 2.54 - 3.38 |
| *G. alogus* | 15 | 6 | 16 | 5.26 - 5.53 |
| *G. alogus* | 25 | 3 | 23 | 3.13 - 4.12 |
| *G. alogus* | 25 | 6 | 23 | 5.57 - 6.19 |
| *G. alogus* | 25 | 9 | 23 | 9.01 - 9.96 |
| *G. alogus* | 25 | 12 | 23 | 11.73 - 11.96 |
| *G. alogus* | 35 | 3 | 33 | 3.80 - 4.29 |
| *G. alogus* | 35 | 6 | 33 | 5.12 - 6.07 |
| *H. antelucana* | 25 | 6 | 23 | 5.57 - 5.97 |
