# Supplementary material for: Tracing Water Sources of Terrestrial Animal Populations with Stable Isotopes: Laboratory Tests with Crickets and Spiders
Source: PLoS One. 2010 Dec 31;5(12):e15696. doi: 10.1371/journal.pone.0015696 (PMC3013119; doi:10.1371/journal.pone.0015696)
Supplement: Table S3 — Results of tests of extraction and processing of water samples for isotope analysis. Raw data are reported for each category of sampling. (DOC) [file pone.0015696.s009.doc]

| Table S3. Results of tests of extraction and processing of water samples for isotope analysis. Raw data are reported for each category of sampling. | | | | | | | | |
| --- | --- | --- | --- | --- | --- | --- | --- | --- |
| Date | extracted? | activated charcoal? | filtered? | flame-sealed? | δ ^2^H | δ ^18^O | difference in δ ^2^H | difference in δ ^18^O |
|  |  |  |  |  |  |  |  |  |
| Feb-10 | N | N | N | N | -60.89 | -8.18 |  |  |
|  |  |  |  |  |  |  |  |  |
| Feb-10 | Y | Y | Y | Y | -62.08 | -8.15 | -1.19 | 0.02 |
| Feb-10 | Y | Y | Y | Y | -62.42 | -8.07 | -1.53 | 0.11 |
| Feb-10 | Y | Y | Y | Y | -60.40 | -7.89 | 0.49 | 0.29 |
| Feb-10 | Y | Y | Y | Y | -60.37 | -8.00 | 0.52 | 0.17 |
| Feb-10 | Y | Y | Y | Y | -60.23 | -7.90 | 0.66 | 0.27 |
| Feb-10 | Y | Y | Y | Y | -60.76 | -8.00 | 0.13 | 0.17 |
| Feb-10 | Y | Y | Y | Y | -62.09 | -8.33 | -1.20 | -0.15 |
| Feb-10 | Y | Y | Y | Y | -61.06 | -8.03 | -0.17 | 0.15 |
| Feb-10 | Y | Y | Y | Y | -60.84 | -7.89 | 0.05 | 0.29 |
| Feb-10 | Y | Y | Y | Y | -60.98 | -8.12 | -0.09 | 0.06 |
| Feb-10 | Y | Y | Y | Y | -60.26 | -8.01 | 0.63 | 0.17 |
| mean |  |  |  |  |  |  | -0.16 | 0.14 |
| SE |  |  |  |  |  |  | 0.24 | 0.04 |
| LCL |  |  |  |  |  |  | -0.63 | 0.06 |
| UCL |  |  |  |  |  |  | 0.31 | 0.22 |
|  |  |  |  |  |  |  |  |  |
| measurement SE | |  |  |  |  |  | 0.6 | 0.2 |
